# Review of Systematic Tendencies in (001), (011) and (111) Surfaces Using B3PW as Well as B3LYP Computations of BaTiO_3_, CaTiO_3_, PbTiO_3_, SrTiO_3_, BaZrO_3_, CaZrO_3_, PbZrO_3_ and SrZrO_3_ Perovskites

**DOI:** 10.3390/ma16247623

**Published:** 2023-12-13

**Authors:** Roberts I. Eglitis, Ran Jia

**Affiliations:** 1 Institute of Solid State Physics, University of Latvia, 8 Kengaraga Str., LV1063 Riga, Latvia; jiaran@jlu.edu.cn; 2 Laboratory of Theoretical and Computational Chemistry, Institute of Theoretical Chemistry, Jilin University, Changchun 130023, China

**Keywords:** B3PW and B3LYP computations, B3LYP functional, B3PW functional, SrTiO_3_ (001) surface, BaTiO_3_ (011) surface, PbTiO_3_ (111) surface, SrZrO_3_ (001) surface, PbZrO_3_ (011) surface, surface energies, polar surfaces

## Abstract

We performed B3PW and B3LYP computations for BaTiO_3_ (BTO), CaTiO_3_ (CTO), PbTiO_3_ (PTO), SrTiO_3_ (STO), BaZrO_3_ (BZO), CaZrO_3_ (CZO), PbZrO_3_ (PZO) and SrZrO_3_ (SZO) perovskite neutral (001) along with polar (011) as well as (111) surfaces. For the neutral AO- as well as BO_2_-terminated (001) surfaces, in most cases, all upper-layer atoms relax inwards, although the second-layer atoms shift outwards. On the (001) BO_2_-terminated surface, the second-layer metal atoms, as a rule, exhibit larger atomic relaxations than the second-layer O atoms. For most ABO_3_ perovskites, the (001) surface rumpling *s* is bigger for the AO- than BO_2_-terminated surfaces. In contrast, the surface energies, for both (001) terminations, are practically identical. Conversely, different (011) surface terminations exhibit quite different surface energies for the O-terminated, A-terminated and BO-terminated surfaces. Our computed ABO_3_ perovskite (111) surface energies are always significantly larger than the neutral (001) as well as polar (011) surface energies. Our computed ABO_3_ perovskite bulk B-O chemical bond covalency increases near their neutral (001) and especially polar (011) surfaces.

## 1. Introduction

Surface as well as interface phenomena, taking place in the ABO_3_ perovskites, including the essence of their (001), (011) and (111) surface and interface electronic properties, are very real problems in present-day physics [1,2,3,4,5,6,7,8,9,10,11,12,13,14,15,16,17,18,19,20,21,22,23,24,25,26]. BaTiO_3_, CaTiO_3_, PbTiO_3_, SrTiO_3_, BaZrO_3_, CaZrO_3_, PbZrO_3_ and SrZrO_3_ perovskites are the members of the well-known ABO_3_ (A = Ba, Ca, Pb, Sr and B = Ti or Zr) perovskite family [27,28,29]. They all have a colossal amount of technologically essential applications [30,31,32,33]. For example, BaTiO_3_ is an important ABO_3_ perovskite ceramic material [34]. It has outstanding dielectric as well as ferroelectric and piezoelectric properties [34]. CaTiO_3_ is extensively employed in electronic ceramic materials [35]. Cubic CTO is also used as a keystone element of Synroc [35]. PbTiO_3_ is one of the worldwide most extensively used piezoelectric and ferroelectric materials for technologically important industrial applications [36,37,38]. SrTiO_3_ is a perovskite material with a wide-ranging spectrum of functional properties as well as physical phenomena [39]. STO is an adaptable substrate for complex oxide electronics engineering [40]. STO possesses excellent superconducting properties [41] as well as impurity- and vacancy-based magnetism [42]. BaZrO_3_ perovskite has several industrially important applications in many quite different technology areas [43]. BZO is extensively used, for example, in solid-oxide fuel cells [44] as well as in wireless systems for communications [45]. CaZrO_3_ is used as an ionic conductor to manufacture solid electrodes for applications in various fuel cells [46]. Moreover, CZO is used also as a key element for different sensor types [47,48]. PbZrO_3_ is a very fascinating perovskite, since it is the end point of the Pb(Zr,Ti)O_3_ alloy system, which is very interesting for numerous important applications in industry [49]. Finally, SrZrO_3_ has a lot of technological applications [50,51,52,53]. For example, in the violet–blue light emission, laser host materials as well as capacitors and oxygen sensors [50,51,52,53]. For that reason, it is clear, that during the last twenty-five years, BTO, CTO, PTO, STO, BZO, CZO, PZO and SZO perovskite (001) surfaces have been comprehensively explored theoretically as well as experimentally around the globe [1,2,3,6,7,11,13,14,16,18,21,23,24,54,55,56,57,58,59,60,61,62,63,64,65,66,67,68,69,70,71,72,73,74,75,76,77,78,79,80,81]. It is worth noting that it is relatively easy to compute the neutral ABO_3_ perovskite (001) surfaces, since they consist of alternating neutral AO and BO_2_ slabs [82,83,84,85]. In contrast, it is very difficult to compute the polar ABO_3_ perovskite (011) and (111) surfaces, since they consist of charged planes of ABO and O_2_ as well as AO_3_ and B, respectively. This is main reason why the ABO_3_ perovskite charged and polar (011) [86,87,88,89,90,91,92,93,94,95] and (111) [96,97,98,99,100,101,102,103,104,105,106,107] surfaces are considerably less studied than their neutral and relatively simple (001) surfaces [1,2,3,6,7,11,13,14,16,18,21,23,24,54,55,56,57,58,59,60,61,62,63,64,65,66,67,68,69,70,71,72,73,74,75,76,77,78,79,80,81,82,83,84,85].

For example, the first B3PW calculations, dealing with polar and charged BaTiO_3_ and PbTiO_3_ (011) surface structures were performed by Eglitis and Vanderbilt in 2007 [1]. Two years later, Zhang et al. [86,87], at ab initio level, computed the electronic and structural characteristics of five different terminations of cubic PTO (110) polar surface [86,87]. The first ab initio computations for the polar SrTiO_3_ (011) surface were carried out by Bottin et al. [88]. They computed the atomic as well as electronic structure of a few (1 × 1) SrTiO_3_ (011) surface terminations [88]. The year after that, Heifets et al. [89] carried out first-principles Hartree–Fock computations for four terminations (Sr, TiO as well as two different O terminations) of the polar STO (011) surface [89]. As the next, Eglitis and Vanderbilt [2] performed hybrid DFT computations for three different terminations (TiO, Sr and O) of the polar and charged STO (011) surface [2]. Two years later, Enterkin et al. [90] described the results for the 3 × 1 extended STO (011) surface structure derived experimentally via transmission electron diffraction [90]. Experimental results, dealing with polar STO (011) surfaces, were also confirmed theoretically using modern ab initio DFT computations as well as scanning tunneling microscopy images [90]. Finally, five years ago, Fleischer et al. [91] experimentally investigated the STO (011) surface using reflectance anisotropy spectroscopy (RAS). World-first ab initio calculations for polar CTO (011) surfaces were performed by Zhang et al. [92]. They constructed four different CTO polar (011) surface terminations and computed the cleavage as well as (011) surface energies [92]. Zhang et al. [92] also computed the CTO (011) surface grand potential as well as the (011) surface electronic and atomic structure [92]. One year later, Eglitis and Vanderbilt, using a B3PW hybrid exchange–correlation functional, investigated three different terminations (TiO, Ca and O) of polar CTO (011) surfaces [3]. They [3] computed the polar CTO (011) surface atomic relaxations, energetics as well as chemical bonding properties for three different (011) surface terminations [3].

Two world-first ab initio simulations for the polar BaZrO_3_ (011) surfaces were performed independently by Heifets et al. [93] and by Eglitis [82] in 2007. Heifets et al. [93] studied the charge redistribution, atomic and electronic structure of several different terminations of BZO (011) surfaces [93]. According to the B3PW computations performed by Eglitis [82], three different terminations of the BZO (011) surface exhibit quite different surface energies. They always (for all three terminations) are considerably larger [82] than for the neutral BZO (001) surfaces. Eglitis and Rohlfing [94] computed the SZO and PZO neutral (001) and polar (011) surface rumplings, relaxations, energetics, charge redistributions as well as the Γ-Γ band gaps [94]. Four years later, Chen et al. [95] investigated the electronic properties and stabilities of SZO (110) (1 × 1) five different polar terminations [95]. Finally, the only existing B3LYP calculations, dealing with polar CZO (110) surfaces, were recently performed by Eglitis and co-workers [10]. 

A quarter of century ago, Hagendorf et al. [96], experimentally investigated the polar BTO (111) surface using scanning tunneling microscopy (STM), X-ray photoelectron spectroscopy (XPS) and low-energy electron diffraction (LEED) methods [96]. Recently, Chun et al. [97], explored the BTO surface (111) termination, using the theoretical ab initio DFT calculations and experimental XPS analysis [97]. First-in-the-world ab initio linearized augmented-plane-wave method (LAPW) calculations for periodic (111) BTO slabs were performed by Cohen [98]. Cohen found [98] that the polar (111) BTO slab is considerably less stable than the BTO neutral (001) slab. In 2015, Eglitis [5,60,99] performed very comprehensive B3LYP calculations for BTO, PTO, CTO, STO, PZO and SZO (111) surfaces. Eglitis found [5] that the polar BTO, PTO, CTO, STO, PZO and SZO (111) surfaces are considerably less stable than the respective neutral (001) and even polar (011) surfaces [5,60,99]. Twenty-five years ago, Haruyama et al. [100] experimentally studied the polar STO (111) surface by means of photoemission spectroscopy [100]. In 1999, Pojani et al. [101], by means of simple semi-empirical HF method, computed the polar STO (111) and (110) surfaces [101]. Recently, Torrelles et al. [102] experimentally detected the surface structure of Ti-terminated STO (111) single crystals [102]. Finally, Marks et al. [103] described the reconstructions of the polar STO (111) surface by means of experimental-transmission electron diffraction, scanning tunneling microscopy as well as theoretical first-principles DFT calculations [103]. Pang et al. [104] performed very comprehensive first-principles computations for four different (1 × 1) polar terminations of PTO (111) surfaces. The electronic and structural properties as well as stabilities of four different polar (1 × 1) PTO (111) terminations were calculated at ab initio level [104]. Liu et al. [105] constructed the stoichiometric as well as nonstoichiometric terminations for polar CTO (111) surfaces [105]. They computed the polar CTO (111) surface electronic structure, grand potential as well as the relevant surface and cleavage energies [105]. Kim et al. [106] explored twenty-two low-indexed BZO (001), (011) as well as (111) surface terminations in order to investigate the Gibbs free energy for their surfaces [106]. Finally, Eglitis [107] performed B3LYP computations for BaO_3_- and Zr-terminated polar BZO (111) surface relaxations and energetics. 

The objective of our review paper was to carry out necessary additional ab initio computations in order to finalize our more-than-20-year-long research work, devoted to ABO_3_ perovskite surfaces. Namely, we report in this place our B3PW and B3LYP computation results for BTO, CTO, PTO, STO, BZO, CZO, PZO, SZO neutral (001) as well as polar (011) and (111) surfaces. We meticulously analyzed B3PW and B3LYP computation results and detected systematic tendencies, typical for all eight of our ab initio computed ABO_3_ perovskite surfaces. Finally, we systematized these common systematic trends in a system, effortlessly approachable worldwide for a comprehensive audience of scientists.

## 2. Computational Details and Surface Models

We performed very comprehensive hybrid density functional theory (DFT) calculations for eight different ABO_3_ perovskite (001), (011) and (111) surfaces by means of the CRYSTAL [108] computer program. The CRYSTAL computer program [108] utilizes Gaussian-type well-localized basis sets (BSs). The BSs for BTO, PTO and STO perovskites were evolved by Piskunov et al. [109]. Almost all computations in this review were executed by means of the B3PW [110,111] or B3LYP [112] hybrid exchange–correlation functionals. It is worth noting that the hybrid exchange–correlation functionals, like B3PW or B3LYP, enable us to reach an outstanding agreement with the experiment [10,75] for the Γ-Γ band gaps of different ABO_3_ perovskites. We executed the reciprocal-space integration for the ABO_3_ perovskite bulk and their surfaces by examining the Brillouin zone, utilizing the 8 × 8 × 8 and 8 × 8 × 1 times, respectively, enlarged Pack Monkhorst grid [113]. The trump card of the CRYSTAL computer program [108] is its ability to compute isolated, two-dimensional slabs, without any unnatural periodicity in the direction *z*, perpendicular to the slab surface. We performed B3PW and B3LYP computations for all eight ABO_3_-type perovskites and their surfaces in high symmetry, cubic structure $(space\;group\;Pm\bar{3}m$) [114,115,116]. 

**Figure 1:**
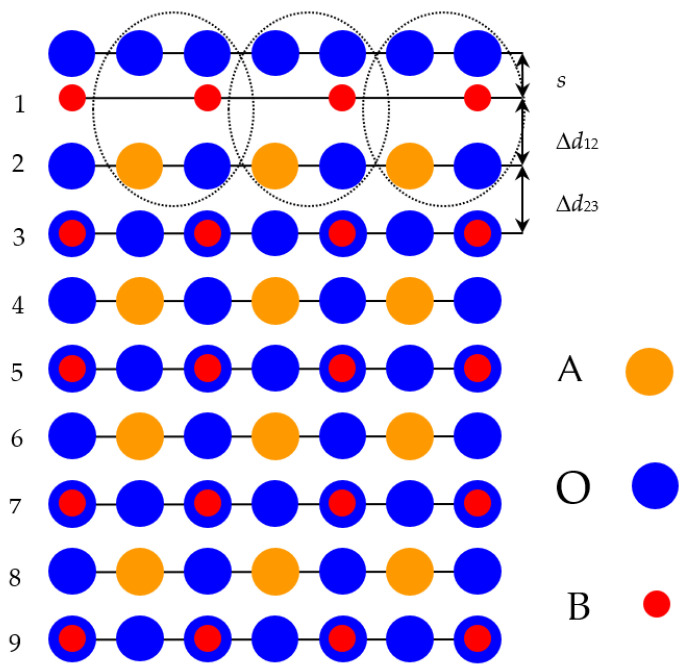
Profile for the BO_2_-terminated (001) surface of ABO_3_-type perovskite accommodating nine layers and containing the definition of the surface rumpling *s* as well as the near-surface interplane distances Δ*d*_12_ and Δ*d*_23_.

**Figure 2:**
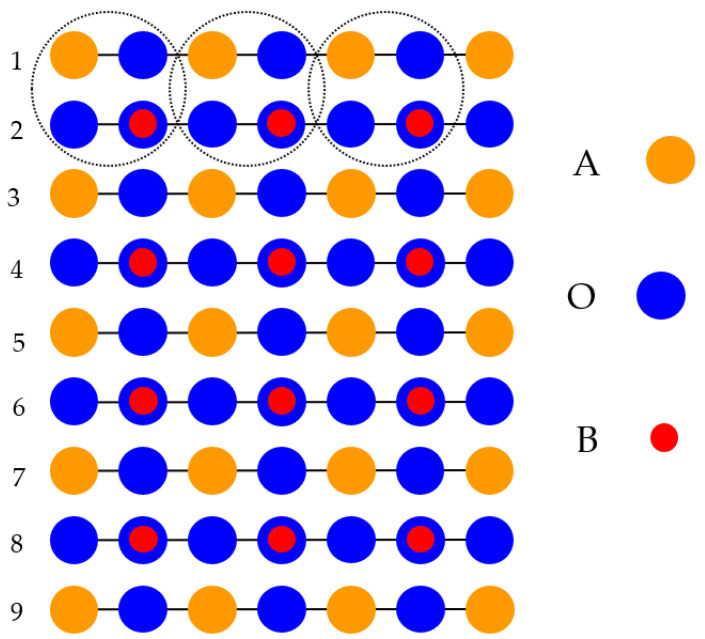
Profile for the AO-terminated (001) surface of ABO_3_-type perovskite accommodating nine layers.

With the goal of simulating the neutral BO_2_-terminated (001) surfaces of ABO_3_-type perovskites [114], we selected symmetrical slabs. These slabs, in our computations, consisted of nine neutral and alternating BO_2_ as well as AO layers (Figure 1 and Figure 2). The first slab was terminated by the BO_2_ planes and was composed of a supercell which accommodated 23 atoms (Figure 1). The second slab was terminated by the AO planes and was composed of a supercell which accommodated 22 atoms (Figure 2). Both these (001) surface slabs are nonstoichiometric. They have unit cell formulas equal to A_4_B_5_O_14_ and A_5_B_4_O_13_, respectively (Figure 1 and Figure 2).

**Figure 3:**
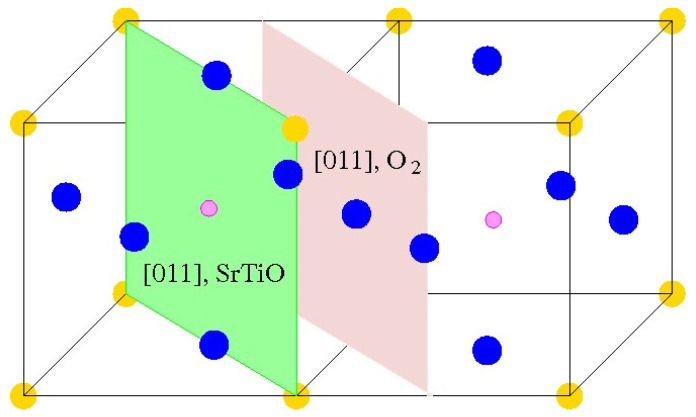
Sketch of the cubic ABO_3_ perovskite construction, containing two (011) cleavage planes, consisting of charged O_2_ as well as ABO (011) surfaces.

**Figure 4:**
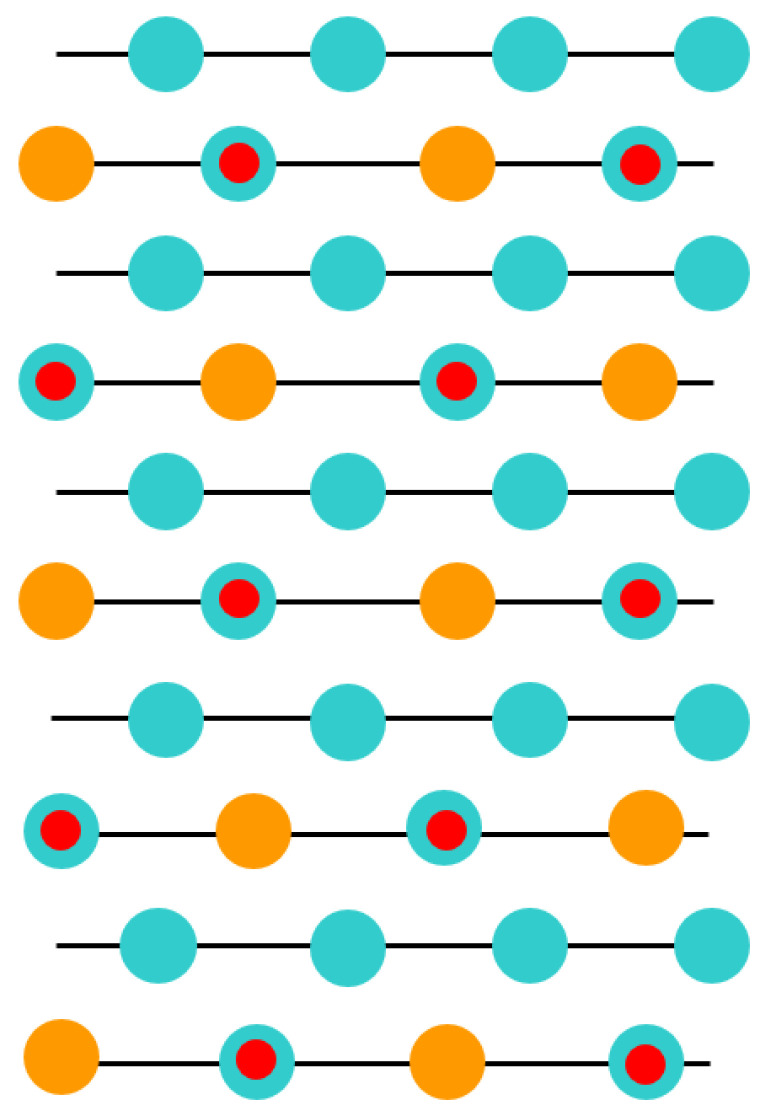
Sketch of the ABO_3_ perovskite (011) surface slab models. Slabs are derived by ABO_3_ perovskite (011) cleavage yielding mixed O_2_ and ABO terminations.

**Figure 5:**
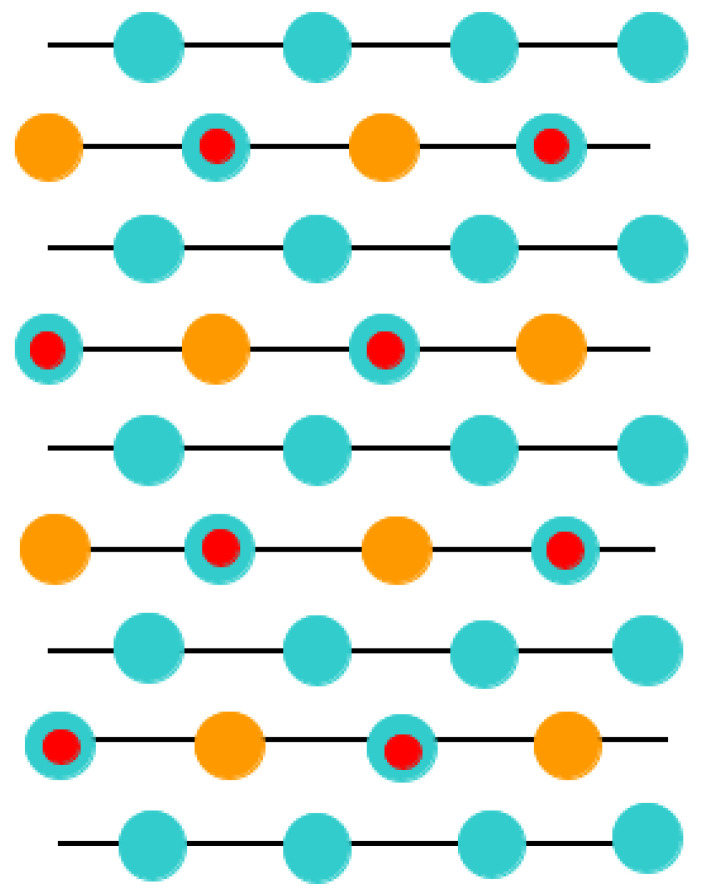
Sketch of the ABO_3_ perovskite (011) surface slab models. Slabs are derived by ABO_3_ perovskite (011) cleavage yielding an O_2_-terminated surface.

**Figure 6:**
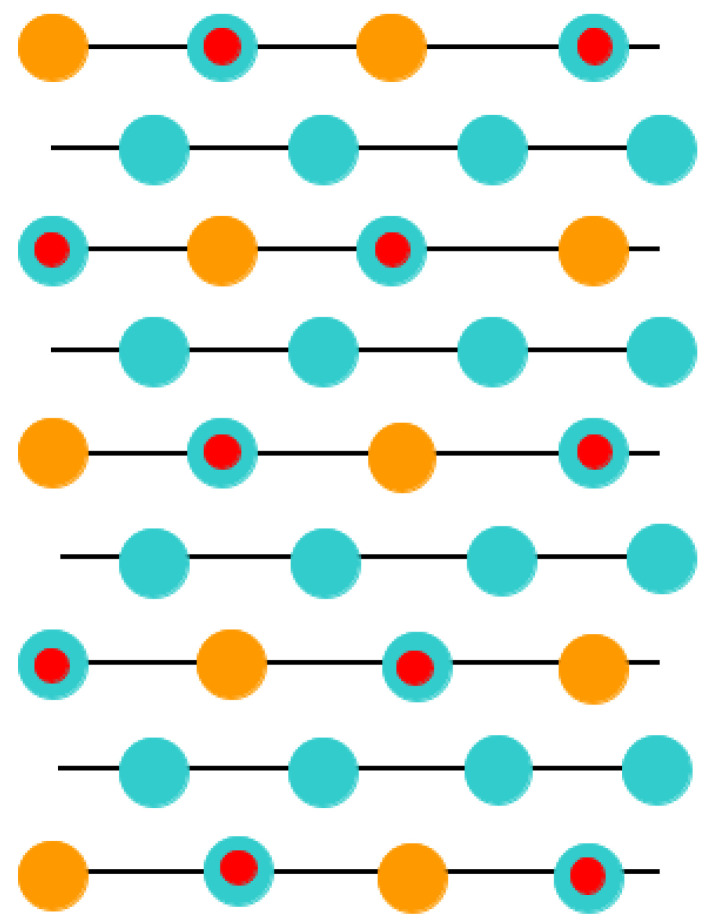
Sketch of the ABO_3_ perovskite (011) surface slab models. Slabs are derived by ABO_3_ perovskite (011) cleavage yielding an ABO-terminated surface.

Just opposite to the (001) cleavage (Figure 1 and Figure 2) of ABO_3_, which produce nonpolar AO and BO_2_ terminations, direct cleavage of ABO_3_-type perovskites, in order to generate (011) surfaces, leads to the production of polar O_2_ as well as ABO surfaces (Figure 3). The ABO_3_ crystal (Figure 3), alongside the [011] crystalic direction, is composed of cyclic planes of O_2_ and ABO units (Figure 3). These two alternating O_2_ and ABO planes (Figure 3) have ionic charges of −4*e* and +4*e*, assuming following constituents as O^2−^, B^4+^ and A^2+^. Therefore, modeling of the ABO_3_ (011) surfaces (Figure 3) precisely, as they are obtained from the pristine crystal cleavage, leads to the following two problematic situations: An infinite macroscopic dipole moment, which is perpendicular to the ABO_3_ perovskite (011) surface (Figure 4), when the slab is terminated by different O_2_ as well as ABO planes (Figure 4) (stoichiometric slab). Infinite charge, in case when the slab is terminated by the same planes (O_2_-O_2_) (Figure 5) or ABO-ABO (Figure 6) (nonstoichiometric slab). Such ABO_3_ perovskite (011) surface terminations (Figure 5 and Figure 6) make the (011) surface unstable [117,118]. 

**Figure 7:**
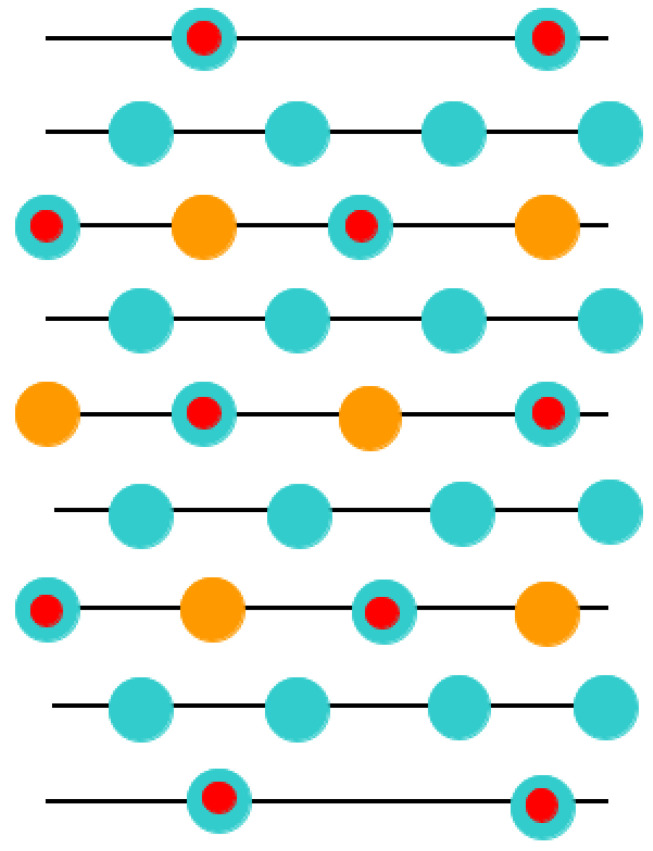
Sketch of the ABO_3_ perovskite (011) surface slab models. Slabs are derived by ABO_3_ perovskite (011) cleavage yielding a BO-terminated surface.

**Figure 8:**
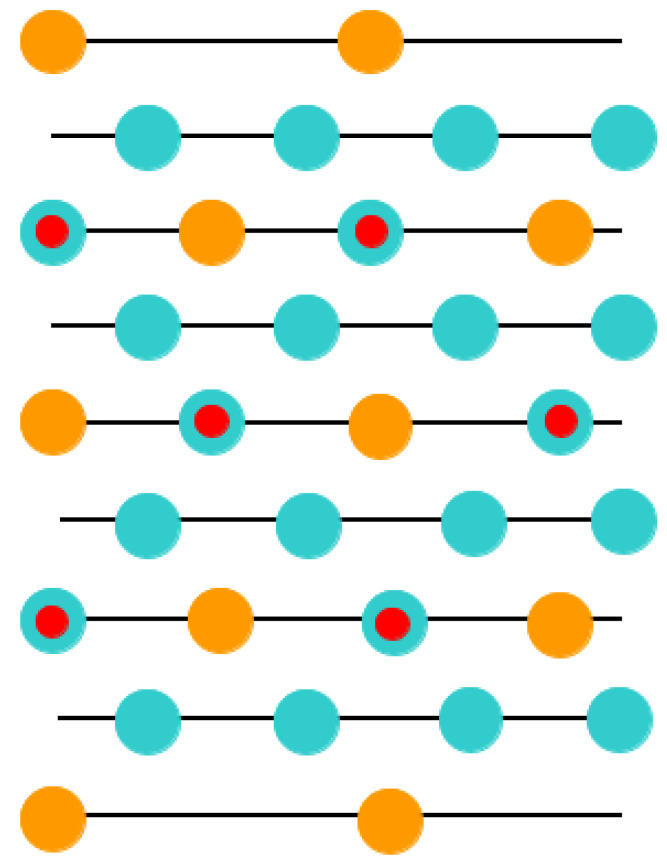
Sketch of the ABO_3_ perovskite (011) surface slab models. Slabs are derived by ABO_3_ perovskite (011) cleavage yielding an A-terminated surface.

**Figure 9:**
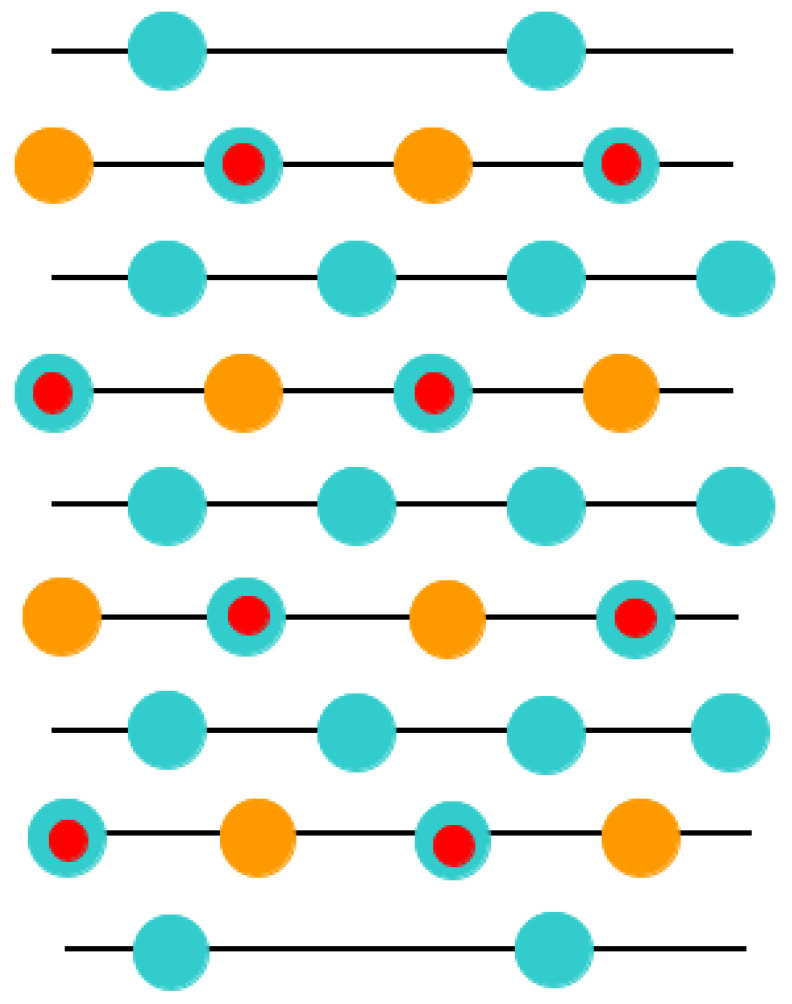
Sketch of the ABO_3_ perovskite (011) surface slab models. Slabs are derived by ABO_3_ perovskite (011) cleavage yielding an O-terminated surface.

This was the key reason why in our ABO_3_-type perovskite (011) surface computations, with the aim of obtaining the neutral (011) slab, we deleted some atoms (Figure 7, Figure 8 and Figure 9). Namely, we deleted the O atom (Figure 9) from the upper as well lower layers of the nine-layer O-O-terminated symmetric nonstoichiometric (011) slab. Thus, we obtain a neutral O-terminated ABO_3_ perovskite (011) slab without any dipole moment perpendicular to the slab surface (Figure 9). Similarly, we deleted both B and O atoms (Figure 8) or an A atom (Figure 7) from the upper and lower layers of the ABO-terminated symmetric nonstoichiometric ABO_3_ perovskite (011) slabs. Thus, we obtain neutral A-terminated (Figure 8) or BO-terminated (Figure 7) ABO_3_ perovskite (011) slabs without any dipole moment perpendicular to their (011) surfaces. Consequently, in our computations, the BO-terminated symmetric, nonstoichiometric (Figure 7) nine-layer (011) slab consisted of a supercell enclosing 21 atoms. The A- (Figure 8) and O- (Figure 9) terminated nonstoichiometric and symmetric ABO_3_ perovskite nine-layer (011) slabs consisted of supercells enclosing 19 and 20 atoms, respectively. 

**Figure 10:**
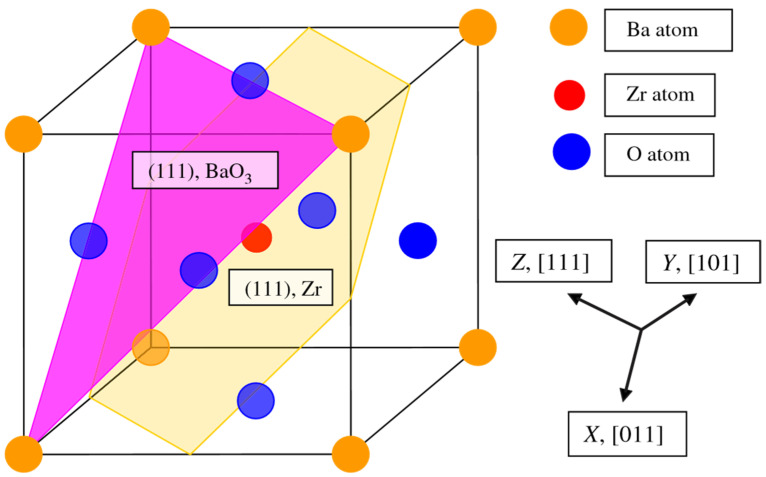
Cubic BZO perovskite structure exhibiting two (111) surface terminations: BaO_3_ and Zr.

**Figure 11:**
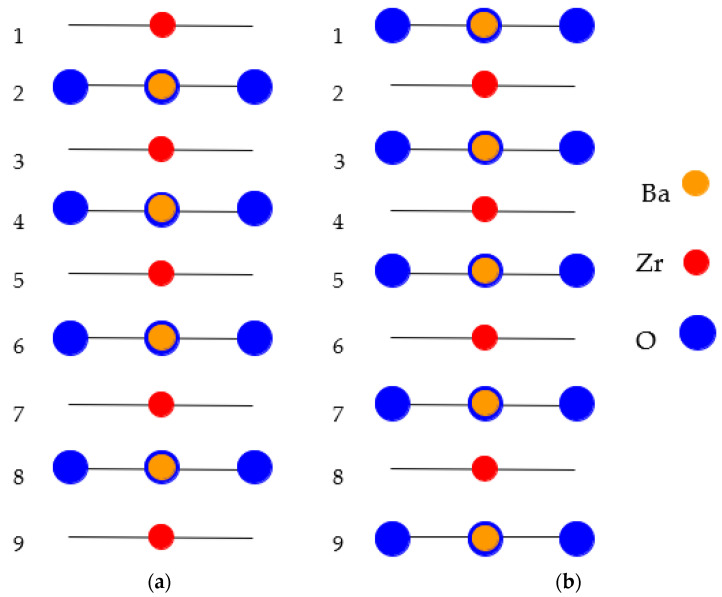
Profile of slab geometries employed to explore BZO (111) surfaces. (**a**) Nonstoichiometric BZO slab containing nine layers with Zr-terminated (111) surfaces. (**b**) Nonstoichiometric BZO slab containing nine layers with BaO_3_-terminated (111) surfaces.

As a further action, the ABO_3_ perovskite polar (111) surfaces will be described by us using BZO as an example (Figure 10 and Figure 11) [107]. In order to compute the polar BZO perovskite (111) surfaces, we employed symmetrical, nonstoichiometric (111) slabs containing nine alternating Zr and BaO_3_ layers (Figure 10 and Figure 11). One of two BZO (111) slabs (Figure 11a) is terminated by Zr planes from both sides. It consists of a supercell accommodating 21 atoms (Figure 11a). The second (111) slab (Figure 11b) is terminated from both sides by BaO_3_ planes. It consists of a supercell accommodating 24 atoms (Figure 11b). Both these Zr- and BaO_3_-terminated BZO (111) slabs are symmetrical and nonstoichiometric (Figure 11). They have the unit-cell formulas Ba_4_Zr_5_O_12_ and Ba_5_Zr_4_O_15_, respectively (Figure 11). As we know from studies dealing, for example, with polar STO and CTO (111) surfaces [99,101,119], a strong electron redistribution happens for such (111) terminations (Figure 11) canceling the polarity. Therefore, such calculations are possible for the Zr- or BaO_3_-terminated BZO (111) surface [99,101,119]. It is worth noting that we used the basis sets for neutral Ba, Zr and O atoms in all our B3LYP computations dealing with polar BaZrO_3_ perovskite (111) surfaces [5,99,107]. 

With the ultimate goal of computing the ABO_3_-type perovskite, for example, the PbZrO_3_ (001) surface energy, we started our B3LYP computations with the cleavage energy calculations for unrelaxed PbO- as well as ZrO_2_-terminated (001) surfaces [1,2,3,94]. Surfaces with both PbO and ZrO_2_ (001) terminations at the same time emerge under the (001) cleavage of the PZO crystal [1,2,3,94]. We suppose that the PZO perovskite cleavage energy is uniformly shared between the created (001) surfaces (Figure 1 and Figure 2) [1,2,3]. In our B3LYP computations, the nine-layer PbO-terminated PZO (001) slab with 22 atoms as well as the nine-layer ZrO_2_-terminated PZO (001) slab, containing 23 atoms, together contain nine bulk unit cells or 45 atoms atoms, thus:

*E*_surf_^unr^(ϑ) = ¼ [*E*_slab_^unr^(PbO) + *E*_slab_^unr^(ZrO_2_) − 9*E*_bulk_], (1)

where ϑ means PbO or ZrO_2_; *E*_slab_^unr^(ϑ) is the SrO- or ZrO_2_-terminated PZO (001) slab energies without relaxation; *E*_bulk_ is the PZO bulk unit cell, containing five atoms, total energy; and the factor of ¼ means that we created four surfaces due the PZO crystal (001) cleavage [1,2,3]. After this, we can compute the relaxation energies for both PbO- and ZrO_2_-terminated PZO (001) slabs [1,2,3,75,82], using the following equation:

*E*_rel_(ϑ) = ½ [*E*_slab_^rel^(ϑ) − *E*_slab_^unr^(ϑ)], (2)

where *E*_slab_^rel^(ϑ) is the (001) slab total energy after geometry relaxation [1,2,3,75,82]. The surface energy is thereby described as a sum of the relevant relaxation as well as cleavage energies:

*E*_surf_(ϑ) = *E*_surf_^unr^(ϑ) + *E*_rel_(ϑ). (3)

With goal of computing the PZO (011) surface energies for the ZrO- and Pb-terminated (011) surfaces, we think about the cleavage of eight PZO bulk unit cells, in order to obtain the ZrO- and Pb-terminated (011) slabs, which contain 21 and 19 atoms. Namely, we split the cleavage energy uniformly among these two surfaces and derive:

*E*_surf_^unr^(ϑ) = ¼ [*E*_slab_^unr^(Pb) + *E*_slab_^unr^(ZrO) − 8*E*_bulk_], (4)

where ϑ indicates Pb or ZrO; *E*_slab_^unr^(ϑ) is our computed total energy for the unrelaxed Pb- or ZrO-terminated PZO (011) slabs; and *E*_bulk_ is our computed PbZrO_3_ perovskite total energy per five-atom bulk unit cell.

In the end, when we cut the PZO perovskite crystal in other way, we obtain two equal O-terminated PZO (011) surface slabs. Each of them contains 20 atoms [1,2,3,82]. This permits us to make our computations less complex, taking into account that the unit cell of the nine-plane O-terminated PZO (011) slab includes four PZO bulk unit cells [1,2,3,82]. Thereby, the O-terminated PZO perovskite (011) surface energy is described as follows:

*E*_surf_(O) = ½ [*E*_slab_^rel^(O) − 4*E*_bulk_], (5)

where *E*_surf_(O) is the O-terminated PZO (011) surface energy, and *E*_slab_^rel^(O) is the relaxed O-terminated PZO (011) slab total energy. In the end, the ABO_3_ perovskite polar (111) surface energy computation details are described by us in Refs. [5,99,107]. 

## 3. Results

### 3.1. B3PW and B3LYP Calculations of ABO_3_ Perovskite Bulk Properties

As a starting point of calculations, we computed the bulk lattice constants for all eight of our considered ABO_3_-type perovskites, using two different hybrid exchange–correlation functionals, B3PW and B3LYP. We compared our theoretically computed ABO_3_ perovskite bulk lattice constants with available experimental data. Namely, our computed bulk lattice constants, using the B3PW hybrid exchange–correlation functional for BTO (4.008 Å) [1], CTO (3.851 Å) [3], PTO (3.936 Å) [1], STO (3.904 Å) [2], BZO (4.234 Å) [82] and SZO (4.155 Å) [75] perovskites are listed in Table 1. Next, we computed the relevant eight ABO_3_ perovskite bulk lattice constants also using the related B3LYP hybrid exchange–correlation functional. Namely, using B3LYP, we obtain the following results for BTO (4.04 Å) [109], CTO (3.851 Å) [99], PTO (3.96 Å) [109], STO (3.94 Å) [109], BZO (4.234 Å) [107], CZO (4.157 Å) [120], PZO (4.220 Å) [94] and SZO (4.195 Å) [94] (Table 1). Experimental ABO_3_-type perovskite bulk lattice constants for our eight computed ABO_3_ perovskites are collected in Table 1 [121,122,123,124,125,126] for comparison purposes.

As we can see from Table 1, both in our computations for ABO_3_ perovskite bulk lattice constant used B3PW and B3LYP hybrid exchange–correlation functionals allows us to achieve fair agreement with the experiment [121,122,123,124,125,126]. For example, the agreement between our B3PW computed SrZrO_3_ bulk lattice constant 4.155 Å [75] and the experimental value of 4.154 Å [126] is simply outstanding. Also, our B3PW computed BTO bulk lattice constant 4.008 Å [1] is in almost perfect agreement with the experimental data of 4.00 Å [121]. In addition, the agreement between our B3LYP computed PbTiO_3_ bulk lattice constant (3.96 Å) [109] and the experimental PbTiO_3_ bulk lattice constant (3.97 Å) [123] is fine. 

Our B3PW or B3LYP computed bulk effective atomic charges *Q* and bond populations *P* for all eight ABO_3_-type perovskites are collected in Table 2. We used the classical Mulliken population analysis [127,128,129,130] in order to describe the effective atomic charges *Q* as well as chemical bond populations *P* for all eight of our B3PW or B3LYP computed ABO_3_-type perovskite materials (Table 2). As we can see from Table 2, our B3PW or B3LYP computed effective atomic charges *Q* [127,128,129,130] are always smaller than those expected from the classical ionic model (+2*e* for A atoms, +4*e* for B atoms as well as −2*e* for O atoms). For example, the A atom effective charges (Table 2) are in the range of only +1.354*e* for the PTO perovskite to +1.880*e* for the SZO perovskite (Table 2). The B atom effective charges are in the range (Table 2) from +2.111*e* for PZO to +2.367*e* for BTO perovskite. The O atom effective charges [127,128,129,130] are between −1.160*e* for PZO perovskite (Table 2) and −1.407*e* for the STO perovskite. Finally, the smallest B-O chemical bond population *P*, according to our B3PW computations, is observed between the Ti-O atom in the CTO perovskite (+0.084*e*), whereas the largest is between the Zr-O atoms in the BZO perovskite (+0.108*e*) (Table 2).

**Figure 12:**
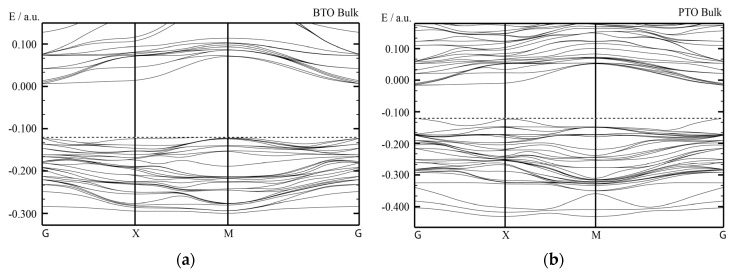
Our hybrid B3PW computed [114] bulk electronic band structure for BTO (**a**) as well as PTO (**b**) perovskites. The dotted lines correspond to the bulk valence band maximum.

**Figure 13:**
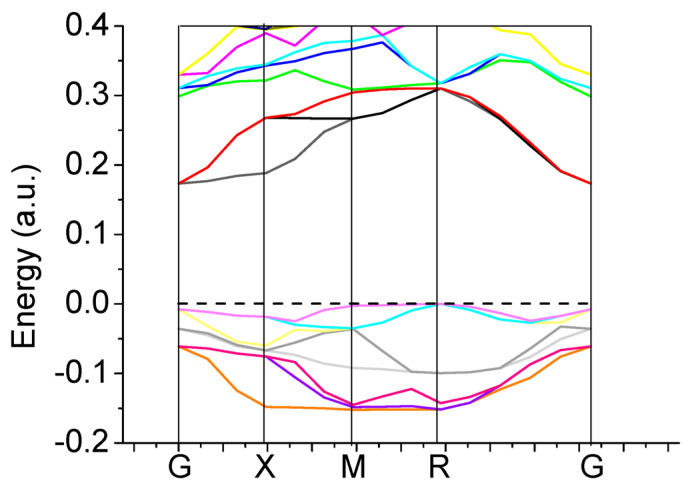
Our hybrid B3PW computed bulk electronic band structure for BZO [75] perovskite.

**Figure 14:**
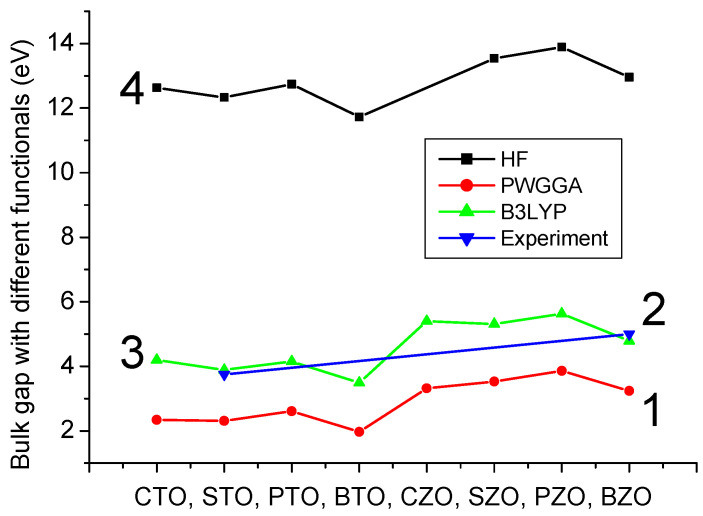
Our computed as well as experimentally measured bulk band gaps at Γ-point for 8 ABO_3_ perovskites obtained using different functionals: (1) PWGGA, (2) experimental data, (3) B3LYP and (4) HF.

Our B3PW computed bulk Γ-Γ band gap for the BTO perovskite is equal to 3.55 eV (Table 3 and Figure 12a). No experimental data exist for the BTO bulk Γ-Γ band gap at the cubic phase. Nevertheless, the related Γ-Γ BTO bulk electronic band structure, measured in the tetragonal towards orthorhombic phase transition temperature [131], identical to 278 K, at contrasting experimental situations, is equivalent to 3.27 or 3.38 eV, respectively. Our B3PW [75] and B3LYP [99] computed CTO bulk Γ-Γ band gaps are almost identical (4.18 eV and 4.20 eV, respectively). Again, there are no experimental data available for the high-temperature cubic CTO phase [75]. It is worth noting that our PWGGA computed CTO bulk Γ-Γ band gap is very small, only 2.34 eV [75], whereas our HF computed CTO bulk Γ-Γ band gap is 5.4 times larger and is equal to 12.63 eV (Table 3). Our B3PW computed PTO bulk Γ-Γ band gap [114] is equal to 4.32 eV (Table 3 and Figure 12b). Our B3PW computed BZO bulk band structure is plotted in Figure 13. Our B3PW computed STO Γ-Γ bulk band gap 3.96 eV [114] is almost in perfect agreement with the available experimental data for the STO cubic phase at Γ-point 3.75 eV [132] (Table 3 and Figure 14). Our B3PW computed BZO bulk band gap at Γ-point is equal to 4.93 eV [75] and is in fair agreement with the relevant experimental data (5.3 eV) [133]. As we can see from Table 3, PWGGA computed BZO bulk band gap at Γ-point is considerably underestimated (3.24 eV), whereas the HF result (12.96 eV) is considerably overestimated regarding the experimental BZO bulk band gap value of 5.3 eV. Finally, for SZO perovskite, our B3PW and B3LYP computed bulk Γ-Γ band gaps almost coincide (5.30 eV and 5.31 eV, respectively) (Table 3) [75,94]. Our B3LYP computation results, dealing with eight ABO_3_-type perovskite bulk Γ-Γ band gaps, are depicted in Figure 14. As we can see from Figure 14, the best possible agreement between the theory and experiment for eight ABO_3_-type perovskite bulk Γ-Γ band gaps is possible to achieve by means of the hybrid exchange–correlation functionals, for example B3PW or B3LYP (Table 3 and Figure 14). The HF method hugely overestimated the Γ-Γ bulk band gaps for all eight our computed ABO_3_ perovskites, whereas the density functional theory based PWGGA functional underestimated them (Figure 14 and Table 3).

### 3.2. ABO_3_ Perovskite (001) Surface Atomic and Electronic Structure

Our hybrid exchange–correlation functional B3LYP or B3PW computation results for the (001) surface atomic relaxations for BO_2_- as well as AO-terminated ABO_3_-type perovskite upper three or two (001) surface layers are recorded in Table 4 and Table 5. 

As it is possible to see from Table 4 and Table 5, the atomic relaxation magnitudes of surface metal atoms A or B, for all eight ABO_3_ perovskite (001) surface upper two layers, are almost always noticeably larger than that for the respective O atoms (Table 4 and Table 5). This leads to a significant surface rumpling *s* for the upper-surface plane (Table 6). The only two deviations from this systematic trend are the ZrO_2_-terminated CZO and SZO (001) surface outermost layers, where the Ca as well as Sr atom inward relaxation magnitudes are smaller than the respective O atom inward relaxation magnitudes (Table 5). The second systematic trend is that for both AO and BO_2_ terminations of all eight ABO_3_ perovskite (001) surfaces, as a rule, all atoms of the first (upper) surface layer relax inwards towards the ABO_3_ perovskite bulk (Table 4 and Table 5). At the same time, all atoms of the second surface layer, for both AO and BO_2_ (001) surface terminations, relax upwards (Table 4 and Table 5). Again, all third-layer atoms, the same as upper-layer atoms, relax inwards, towards the ABO_3_ perovskite bulk (Table 4 and Table 5). There are only three exceptions to this systematic trend (Table 4 and Table 5). Namely, TiO_2_-terminated PTO (001) surface upper-layer O atom relaxes upwards by +0.31% of *a*_0_ (Table 5); SrO-terminated STO (001) surface upper-layer O atom relaxes upwards by +0.84% of *a*_0_, whereas the second-layer O atom on the SrO-terminated SrZrO_3_ (001) surface relaxes inwards by a very small relaxation magnitude equal to −0.05% of *a*_0_ (Table 4).

B3PW computed [1,2,3,134,135] as well as experimental [136,137] results, dealing with ABO_3_-type perovskite titanates BTO, CTO, PTO and STO, are collected in Table 6. As we can see from Table 6, our hybrid B3PW computation results [2] for STO (001) surfaces are in fair correspondence with the earlier LDA computation results carried out by Meyer et al. [134]. Namely, both computations, our B3PW [2] as well as those LDA computations performed by Meyer et al. [134], provide the same sign for the changes in interlayer distances Δ*d*_12_ and Δ*d*_23_ [2,134] (Table 6). Moreover, our B3PW computed [2] surface rumplings *s* for SrO as well as TiO_2_-terminated STO (001) surfaces are in fair agreement with the actual LEED [136] as well as RHEED [137] experimental measurements. Nonetheless, our B3PW [2] and LDA [134] computed interlayer distance changes Δ*d*_12_ and Δ*d*_23_ fail to agree with the LEED [136] experimental measurements for the TiO_2_-terminated STO (001) surface. It is worth noting that LEED [136] and RHEED [137] (Table 6) experimental measurements fail to agree concerning the sign of Δ*d*_12_ for the SrO-terminated STO (001) surface. Also, for the TiO_2_-terminated STO (001) surface, LEED [136] and RHEED [137] experiments disagree regarding the sign of the interlayer distance Δ*d*_23_. As we can see from Table 6, our B3LYP [94] as well as Wang et al.’s [138] LDA and GGA computed surface rumpling *s* and relative interlayer displacements Δ*d*_12_ and Δ*d*_23_ for the SrO-terminated SZO (001) surface are in fair agreement with each other. In addition, our B3LYP [94] and Wang et al.’s [138] computed interlayer distances Δ*d*_12_ and Δ*d*_23_ are in good agreement with each other for the ZrO_2_-terminated SZO (001) surface. The agreement between our B3LYP [94] and Wang et al.’s [138] LDA computed surface rumpling *s* for the ZrO_2_-terminated SZO (001) surface (−0.72% of *a*_0_ and −0.7% of *a*_0_) is almost perfect. Unfortunately, the surface rumpling *s*, computed by Wang et al., using the GGA exchange–correlation functional [138] for the ZrO_2_-terminated SZO (001) surface has a different sign of +0.3% of *a*_0_ (Table 6). 

**Figure 15:**
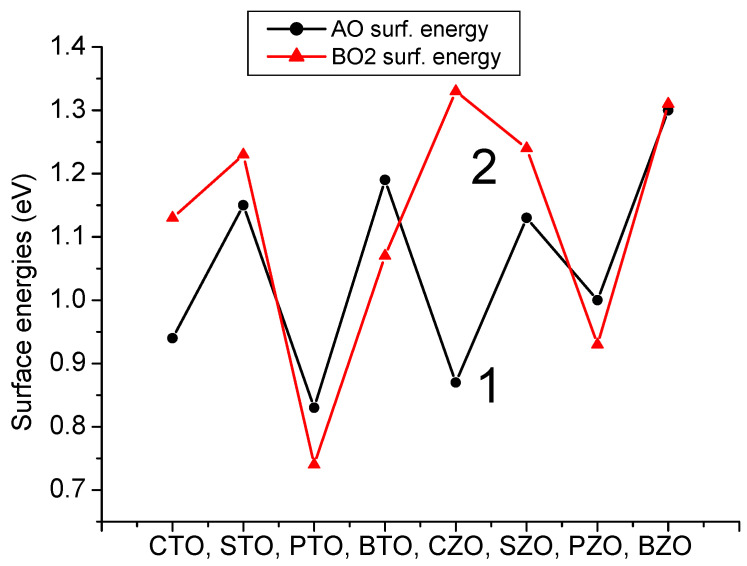
Our B3PW or B3LYP computed AO (1) as well as BO_2_-terminated (2) (001) surface energies (in eV per surface cell) for CTO, STO, PTO, BTO, CZO, SZO, PZO and BZO perovskites.

As we can see from Table 7 and Figure 15, our B3PW or B3LYP computed eight ABO_3_-type perovskite (001) surface energies are always around 1 eV. Namely, our largest computed (001) surface energy is for the ZrO_2_-terminated CaZrO_3_ (001) surface (1.33 eV) [120], whereas the smallest is for the TiO_2_-terminated PbTiO_3_ (001) surface (0.74 eV) [1]. The smallest energy difference, according our B3PW computations, is for the BaZrO_3_ ZrO_2_- (1.31 eV) and BaO− (1.30 eV) terminated (001) surfaces [82]. The largest (001) surface energy difference, according to our B3LYP hybrid exchange–correlation functional computations, is for the CaZrO_3_ perovskite ZrO_2_− (1.33 eV) and CaO− (0.87 eV) terminated (001) surfaces (Table 7 and Figure 10) [120]. It is worth noting that according to the calculation results, the surface energies of the nonpolar BO_2_-terminated (001) surface was slightly smaller for the BTO, PTO and PZO perovskites; thus, it is more stable (Table 7 and Figure 15).

**Figure 16:**
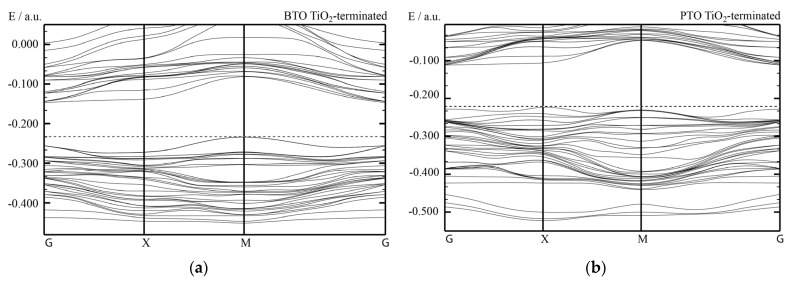
Our B3PW simulated electronic band structure for TiO_2_-terminated ABO_3_ perovskite (001) surfaces of (**a**) BTO and (**b**) PTO.

**Figure 17:**
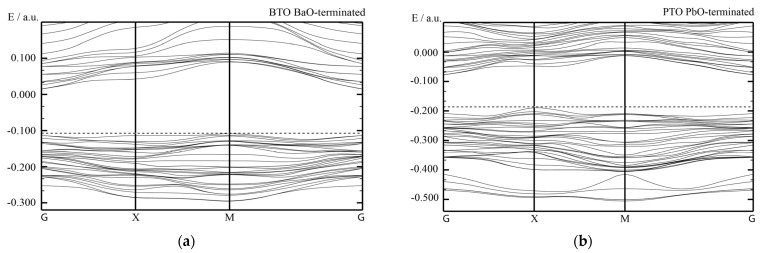
Our B3PW simulated electronic band structures for AO-terminated ABO_3_ perovskite (001) surfaces of (**a**) BTO and (**b**) PTO.

**Figure 18:**
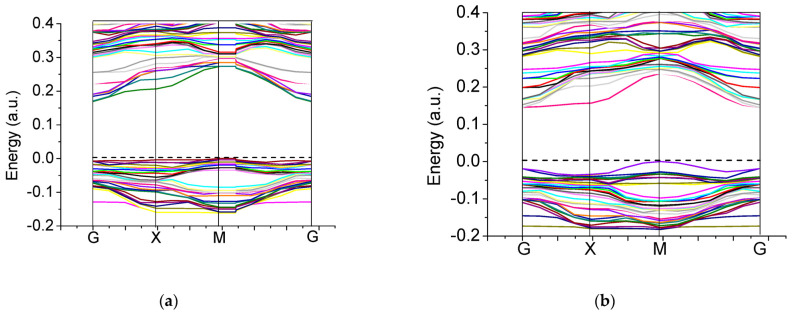
Our B3PW simulated electronic band structures for BaO- (**a**) as well as ZrO_2_-terminated (**b**) BaZrO_3_ (001) surfaces.

**Figure 19:**
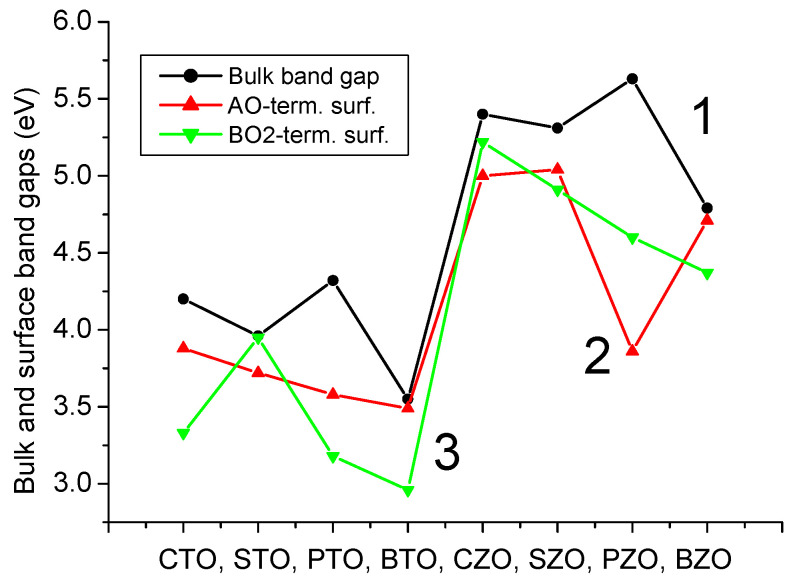
Our computed bulk (1) and AO- (2) as well as BO_2_-terminated (3) (001) surface Γ-Γ electronic band gaps for 8 ABO_3_ perovskites by means of B3LYP or B3PW functionals.

Our B3PW computed electronic bulk band structures for BTO, PTO as well as BZO perovskites are illustrated in Figure 12 and Figure 13. Our B3PW computed TiO_2_-terminated electronic (001) surface band structures for BTO and PTO are depicted in Figure 16a,b, whereas the AO-terminated BTO and PTO (001) surfaces are depicted in Figure 17a,b. Our B3PW computed electronic band structures for BaO- (a) and ZrO_2_- (b) terminated BZO (001) surfaces are illustrated in Figure 18. Our computed Γ-Γ band gap numerical values for all eight of our computed ABO_3_ perovskite bulk as well as their BO_2_- and AO-terminated (001) surfaces are collected in Table 8. As we can see from Table 3 and Table 8, our B3PW computed STO Γ-Γ bulk band gap (3.96 eV) [114] is in an excellent agreement with the experimentally detected STO bulk Γ-Γ band gap (3.75 eV) [133]. Also, for the BZO perovskite Γ-Γ bulk band gap, the agreement between our B3PW computation result (4.93 eV) [75] and the experiment (5.3 eV) [133] is fine (Table 3 and Table 8). The key effect there, as we can see from Table 8 and Figure 19, is that the ABO_3_ perovskite bulk Γ-Γ band gap, for all eight of our B3PW or B3LYP computed ABO_3_ perovskites, is always reduced near their AO- and BO_2_-terminated (001) surfaces. For example, our B3PW computed BZO bulk Γ-Γ band gap (4.93 eV) (Figure 8) is reduced near the BZO ZrO_2_-terminated (001) surface (4.48 eV) as well as near the AO-terminated BZO (001) surface (4.82 eV) (Table 8 and Figure 18 and Figure 19). Also, for all of our other eight computed ABO_3_ perovskites, the situation is similar, regarding the reduction of the ABO_3_ perovskite bulk Γ-Γ band gap near their (001) surfaces (Figure 14 and Table 8). For example, our B3PW computed BTO bulk Γ-Γ band gap (Figure 12a) (3.55 eV) (Table 8) is also reduced near the BaO-terminated BTO (001) surface (3.49 eV) (Figure 17a) and TiO_2_-terminated BTO (001) surface (2.96 eV) (Figure 16a and Figure 19). 

**Figure 20:**
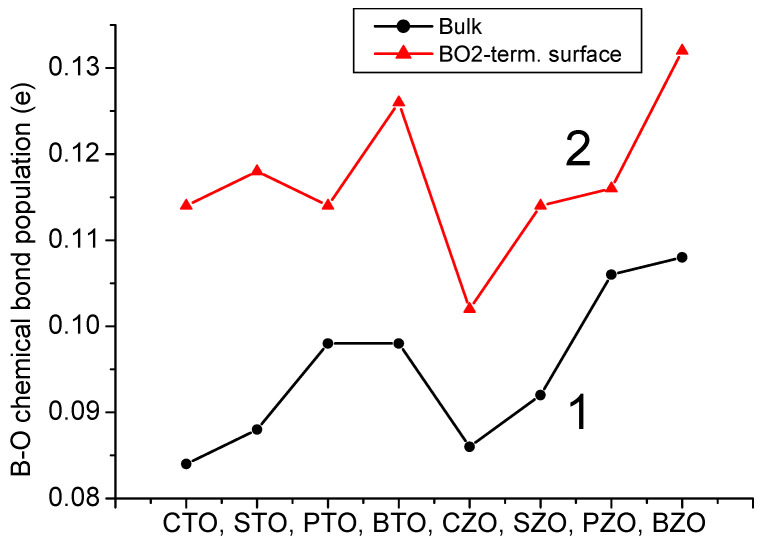
Our B3PW or B3LYP computed bulk (1) as well as BO_2_-terminated (2) (001) surface B-O bond populations for BTO, CTO, PTO, STO, BZO, CZO, PZO and SZO perovskites.

As we can see from Table 9 and Figure 20, for all eight of our B3LYP or B3PW computed ABO_3_ perovskites, we can observe the significant increase in the B-O chemical bond covalency near their BO_2_-terminated (001) surfaces, in comparison with bulk. For example, the largest Ti-O chemical bond population increase by 0.30*e*, according to our B3PW computations, is observed for the CTO and STO perovskites, namely, from 0.084*e* and 0.088*e*, respectively, for their bulk to 0.114*e* and 0.118*e*, respectively, near their TiO_2_-terminated (001) surfaces [2,3]. Just opposite, the smallest B-O chemical bond population increase is observed for the PbZrO_3_ perovskite [94]. Namely, the PbZrO_3_ perovskite Zr-O chemical bond population increased from 0.106*e* (bulk case) to 0.116*e* near the ZrO_2_-terminated PbZrO_3_ (001) surface [94] (Table 9 and Figure 20). 

### 3.3. ABO_3_ Perovskite (011) Surface Atomic and Electronic Structure

**Figure 21:**
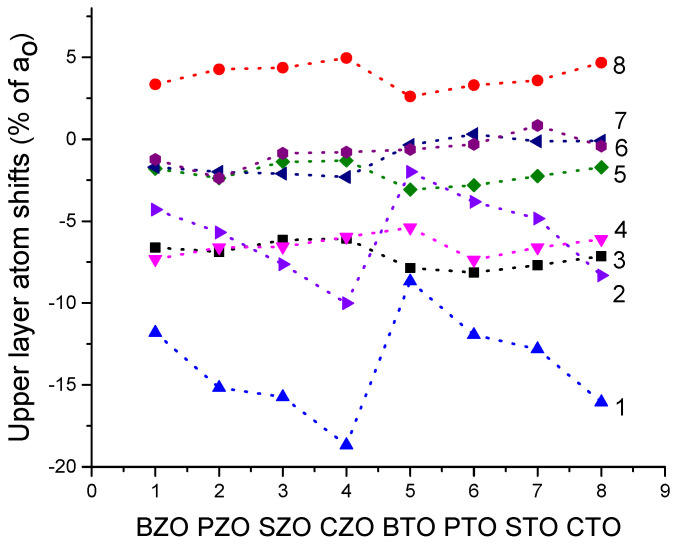
Our computed upper-layer atom shifts for 8 ABO_3_ perovskite BO-, A- and O-terminated (011) and also AO- and BO_2_-terminated (001) surfaces. Line 1, A-term. (011) surface A atom relaxation. Line 2, AO-term. (001) surface, A atom. Line 3, BO-term. (011) surface, B atom. Line 4, O-terminated (011) surface. Line 5, BO_2_-terminated (001) surface, B atom. Line 6, AO-term. (001) surface, O atom relaxation. Line 7, BO_2_-term. (001) surface, O atom relaxation. Line 8, BO-terminated (011) surface, O atom relaxation.

As we can see from Table 10 and Figure 21, for all eight of our B3LYP or B3PW computed ABO_3_ perovskites, the systematic tendency is that for all three of their BO-, A- and O-terminated (011) surfaces, all upper-layer atoms relax inwards. The only exception to this systematic trend is upward relaxation of BO-terminated (011) surface upper-layer O atoms for all eight of our computed ABO_3_ perovskites (Table 10 and Figure 21). 

It is worth noting that the biggest relaxation magnitude between all upper-layer ABO_3_ perovskite (011) surface atoms, for all three possible (011) surface terminations, demonstrates the Ca-terminated surface Ca atom shifting inwards by −18.67% of *a*_0_ (Table 10 and Figure 16) [10]. It is around three times bigger than the displacement magnitudes for the Zr atom (+6.06% of *a*_0_) on the ZrO-terminated as well as O atom (+5.97% of *a*_0_) on the O-terminated CZO (011) surfaces (Table 10 and Figure 21). 

**Figure 22:**
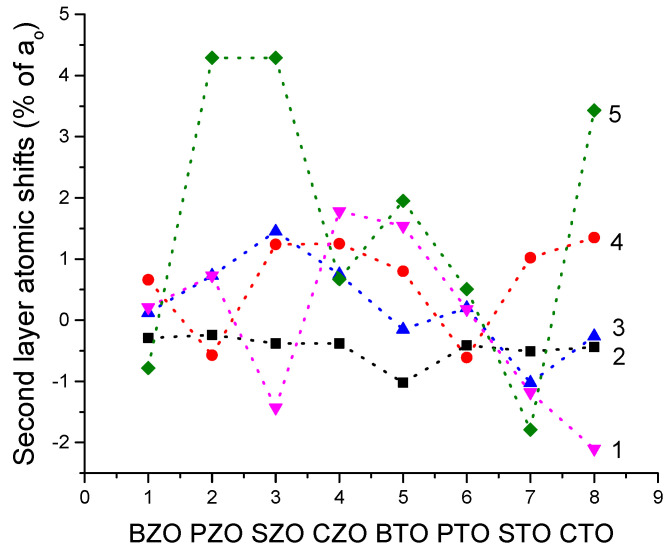
Our B3PW or B3LYP computed second-layer atom relaxation magnitudes. Line 1-our computed O-term. (011) surface A atom shifts. Line 2-BO-term. (011) surface O atom shifts. Line 3-O-term. (011) surface B atom shifts. Line 4-A-term. (011) surface O atom shifts. Line 5-O-term. (011) surface O atom shifts.

As we can see from Table 11 and Figure 22, all our B3LYP computed second-layer O-, Ca- and ZrO-terminated CZO (011) surface atoms relax upwards. The only exception to this systematic trend is the second-layer O atom on the ZrO-terminated CZO (011) surface, which relax inwards (Table 11 and Figure 22). It is worth noting that such systematic trend, mainly upward shift of the second-layer atoms on the A-, O- and BO-terminated (011) surfaces, is quite common for all eight of our computed ABO_3_ perovskites (Table 11 and Figure 22). Namely, according to our B3PW or B3LYP computations for eight ABO_3_ perovskite (011) surface, all second-layer atoms, located on three different (011) terminations, shift upwards 23 atoms, but relax inwards only 17 atoms (Figure 22 and Table 11). 

**Figure 23:**
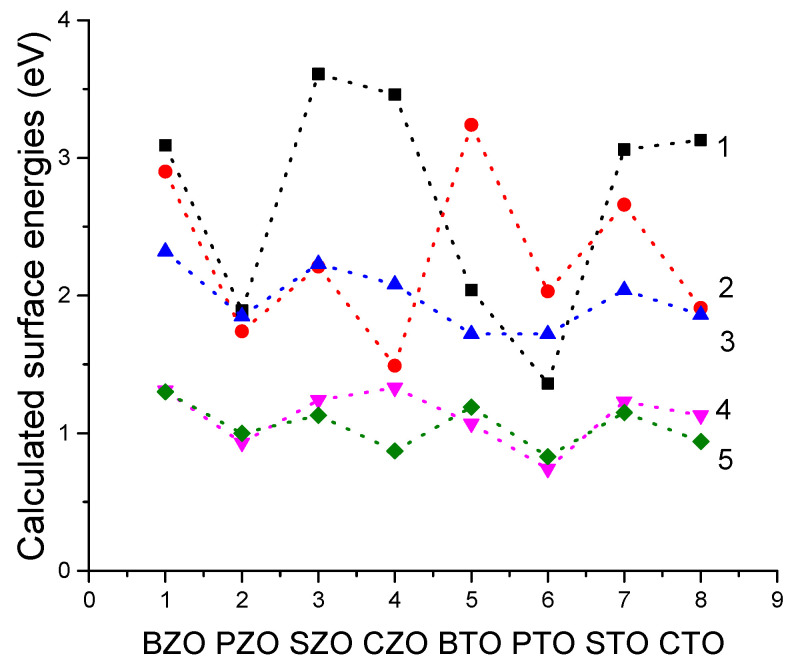
Our B3LYP or B3PW computed ABO_3_ surface energies (in eV) for BO- (1), A- (2), O-terminated (3) (011) and also BO_2_- (4) and AO-terminated (5) (001) surfaces.

As we can see from Table 12 and Figure 23, our B3LYP or B3PW computed ABO_3_ perovskite A-, O- or BO-terminated polar (011) surface energies are always larger than the ABO_3_ perovskite neutral BO_2_- or AO-terminated (001) surface energies. According to our B3LYP computations, the largest ABO_3_ perovskite (011) surface energy is for the ZrO-terminated SZO (011) surface (3.61 eV) (Figure 23 and Table 12). The smallest surface energy between all BO-terminated ABO_3_ perovskite (011) surfaces is for the TiO-terminated PTO (011) surface, only 1.36 eV. This energy (1.36 eV) is only slightly larger than the ZrO_2_-terminated CaZrO_3_ (001) surface energy (1.33 eV). Nevertheless, according to our B3PW or B3LYP computations, the BO-, O- or A-terminated polar ABO_3_ perovskite (011) surface energies are always larger than the neutral BO_2_- or AO-terminated ABO_3_ perovskite (001) surface energies (Figure 19 and Table 12).

**Figure 24:**
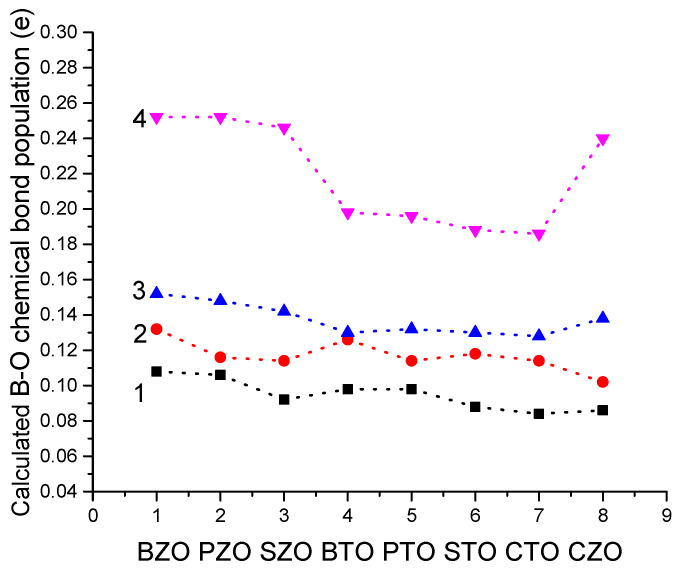
Our B3LYP or B3PW computed B-O chemical bond populations for ABO_3_ perovskite bulk (1), BO_2_-terminated (001) surfaces (2), as well as for BO-terminated (011) surfaces, B(I)-O(I) (3) and B(I)-O(II) (4).

As it is possible to see from Table 13 and Figure 24, according to our B3LYP or B3PW computation results for eight ABO_3_ perovskites, the B-O chemical bond population is the smallest for the ABO_3_ perovskite bulk. The B-O chemical bond population is increased near the BO_2_-terminated (001) surface regarding the bulk value and is in the range of +0.102*e* for the CZO perovskite to 0.132*e* for the BZO perovskite. An even larger B-O chemical bond population is near the BO-terminated (011) ABO_3_ perovskite surface. Namely, the plane B(I)-O(I) chemical bond population for all eight of our computed ABO_3_ perovskites is in the range of 0.128*e* for the CTO perovskite to 0.152*e* for the BZO perovskite (Table 13 and Figure 24). Finally, as we can see from Table 13 and Figure 24, the ultimately largest B-O chemical bond population, according to our B3LYP or B3PW computations for eight ABO_3_ perovskites, is for the BO-terminated (011) surface B(I)-O(II) chemical bond population, in the direction perpendicular to the BO-terminated (011) surface. It is in the range of 0.186*e* for the CTO perovskite to 0.252*e* for the PZO and BZO perovskites (Figure 24 and Table 13). 

### 3.4. ABO_3_ Perovskite (111) Surface Atomic and Electronic Structure

As it is possible to see from Table 14, according to our B3LYP computation results for seven ABO_3_ perovskites, all atoms on the B-terminated ABO_3_ perovskite (111) surface relax inwards. The upper-layer B atom relaxation magnitudes (Table 14) are rather strong, ranging from −3.58% of *a*_0_ for the STO perovskite to −11.19% of *a*_0_ for the BTO perovskite. It is worth noting that almost all second-layer A atoms on the B-terminated ABO_3_ perovskite (111) surface relax inwards. In general, they exhibit very large relaxation magnitudes, for example, −14.02% of *a*_0_ for the second-layer Ca atom on the Ti-terminated CaTiO_3_ (111) surface (Table 14). As we can see from Table 15, according to our B3LYP computations, most of AO_3_-terminated ABO_3_ perovskite upper-layer atoms also relax inwards. Nevertheless, their relaxation magnitudes are considerably smaller than for the upper-layer atom inward relaxation magnitudes on the B-terminated ABO_3_ perovskite upper layer (Table 14 and Table 15). 

As it is possible to see from Table 16, according to our B3LYP computation results for seven ABO_3_ perovskites, the B-terminated ABO_3_ perovskite (111) surface energies are always smaller than the respective AO_3_-terminated ABO_3_ perovskite (111) surface energies. The B-terminated ABO_3_ perovskite (111) surface energies (Table 16) are in the energy range of 4.18 eV for the Ti-terminated CaTiO_3_ (111) surface to 8.19 eV for the Zr-terminated CaZrO_3_ (111) surface. The AO_3_-terminated ABO_3_ perovskite (111) surface energies are in the range of 5.86 eV for the CaO_3_-terminated CaTiO_3_ (111) surface to 9.62 eV for the CaO_3_-terminated CaZrO_3_ (111) surface (Table 16). 

## 4. Conclusions

We performed B3PW and B3LYP computations for BTO, CTO, PTO, STO, BZO, CZO, PZO and SZO perovskite neutral (001) along with polar (011) and (111) surfaces. For the neutral AO- as well as BO_2_-terminated (001) surfaces, in most cases, all upper-layer atoms relax inwards, although the second-layer atoms shift outwards. There are only three exceptions to this systematic trend (Table 4 and Table 5). Namely, upward relaxation of TiO_2_-terminated PTO (001) surface upper-layer O atom by (+0.31% of *a*_0_) (Table 5). On the (001) BO_2_-terminated surface, the second-layer metal atoms, as a rule, exhibit larger atomic relaxations than the second-layer O atoms. For most ABO_3_ perovskites, the (001) surface rumpling *s* is bigger for the AO- than BO_2_-terminated surfaces. In contrast, the surface energies, for both (001) terminations, are practically identical. Nevertheless, for the BTO, PTO and PZO perovskites, the BO_2_-terminated (001) surface has a slightly smaller surface energy, and therefore, it is more stable. In contrast, for CTO, STO, CZO and SZO perovskites, the AO-terminated (001) surface has a slightly smaller surface energy, and therefore, it is slightly more stable. Conversely, different (011) surface terminations exhibit quite different surface energies for the O-terminated, A-terminated, and BO-terminated surfaces. Our computed ABO_3_ perovskite (111) surface energies are always significantly larger than the neutral (001) and polar (011) surface energies. Our computed ABO_3_ perovskite bulk B-O chemical bond covalency increase near their neutral (001) and especially polar (011) surfaces. It is worth noting that for the (011) surfaces, in the plane B(I)-O(I) chemical bond population for all eight of our computed ABO_3_ perovskites is in the range of 0.128*e* for the CTO perovskite to 0.152*e* for the BZO perovskite (Table 13 and Figure 24). Finally, as we can see from Table 13 and Figure 24, the ultimately largest B-O chemical bond population, according to our B3LYP or B3PW computations for eight ABO_3_ perovskites, is for the BO-terminated (011) surface B(I)-O(II) chemical bond population, in the direction perpendicular to the BO-terminated (011) surface. It is in the range of 0.186*e* for the CTO perovskite to 0.252*e* for the PZO and BZO perovskites (Figure 24 and Table 13). 

## Figures and Tables

**Table 1:** Computed ABO_3_ perovskite bulk lattice constants [1,2,3,75,82,94,99,107,109,120] (in Å) by means of the B3PW or B3LYP method. The experimentally detected ABO_3_ perovskite bulk lattice constants [121,122,123,124,125,126] are listed for comparison purposes.

ABO_3_-Type Perovskite	B3PW	B3LYP	Experiment
BaTiO_3_	4.008 [1]	4.04 [109]	4.00 [121]
CaTiO_3_	3.851 [3]	3.851 [99]	3.8967 [122]
PbTiO_3_	3.936 [1]	3.96 [109]	3.97 [123]
SrTiO_3_	3.904 [2]	3.94 [109]	3.89 [121]
BaZrO_3_	4.234 [82]	4.234 [107]	4.199 [124]
CaZrO_3_	-	4.157 [120]	-
PbZrO_3_	-	4.220 [94]	4.1614 [125]
SrZrO_3_	4.155 [75]	4.195 [94]	4.154 [126]

**Table 2:** BTO, CTO, PTO, STO, BZO, CZO, PZO and SZO perovskite bulk effective atomic charges *Q* (in *e*) and bond populations *P* (in *e*) computed using the hybrid B3PW or B3LYP exchange–correlation functionals [1,2,3,82,94].

		BTO	CTO	PTO	STO	BZO	CZO	PZO	SZO
Ion	Prop.	B3PW	B3PW	B3PW	B3PW	B3PW	B3LYP	B3LYP	B3LYP
A	*Q*	+1.797	+1.782	+1.354	+1.871	+1.815	+1.787	+1.368	+1.880
	*P*	−0.034	+0.006	+0.016	−0.010	−0.012	+0.014	+0.030	+0.002
O	*Q*	−1.388	−1.371	−1.232	−1.407	−1.316	−1.310	−1.160	−1.351
	*P*	+0.098	+0.084	+0.098	+0.088	+0.108	+0.086	+0.106	+0.092
B	*Q*	+2.367	+2.330	+2.341	+2.351	+2.134	+2.144	+2.111	+2.174

**Table 3:** B3LYP, B3PW, HF and PWGGA computed Γ-Γ band gaps (in eV) for eight ABO_3_ perovskites. Experimental bulk Γ-Γ band gaps at ABO_3_ perovskite cubic phase are listed for comparison purposes.

Perovskite	Theoretical Method	Bulk Band Gap	Experiment
BaTiO_3_	B3PW	3.55 [114]	No data for cubic phase
CaTiO_3_	B3PW	4.18 [75]	No data for cubic phase
	B3LYP	4.20 [99]	
	PWGGA	2.34 [75]	
	HF	12.63 [75]	
PbTiO_3_	B3PW	4.32 [114]	No data for cubic phase
SrTiO_3_	B3PW	3.96 [114]	3.75 [132]
BaZrO_3_	B3PW	4.93 [75]	5.3 [133]
	B3LYP	4.79 [107]	
	PWGGA	3.24 [75]	
	HF	12.96 [75]	
CaZrO_3_	B3LYP	5.40 [120]	No data for cubic phase
PbZrO_3_	B3LYP	5.63 [94]	No data for cubic phase
SrZrO_3_	B3PW	5.30 [75]	No data for cubic phase
	B3LYP	5.31 [94]	
	PWGGA	3.53 [75]	
	HF	13.54 [75]	

**Table 4:** B3LYP or B3PW computed relaxations of atoms (% of *a*_0_) for the AO-terminated (001) surfaces of eight ABO_3_ perovskites [1,2,3,75,82,94,120].

ABO_3_ Perovsk.		BTO	CTO	PTO	STO	BZO	CZO	PZO	SZO
Termin., (001)		AO	AO	AO	AO	AO	AO	AO	AO
Layer	Ion	B3PW	B3PW	B3PW	B3PW	B3PW	B3LYP	B3LYP	B3LYP
1	A	−1.99	−8.31	−3.82	−4.84	−4.30	−10.01	−5.69	−7.63
	O	−0.63	−0.42	−0.31	+0.84	−1.23	−0.79	−2.37	−0.86
2	B	+1.74	+1.12	+3.07	+1.75	+0.47	+1.11	+0.57	+0.86
	O	+1.40	+0.01	+2.30	+0.77	+0.18	+0.01	+0.09	−0.05
3	A	-	-	-	-	−0.01	−2.60	−0.47	−1.53
	O	-	-	-	-	−0.14	−0.48	−0.47	−0.45

**Table 5:** B3LYP or B3PW computed relaxations of atoms (% of *a*_0_) for the BO_2_-terminated (001) surfaces of eight ABO_3_ perovskites [1,2,3,75,82,94,120].

ABO_3_ Perovsk.		BTO	CTO	PTO	STO	BZO	CZO	PZO	SZO
Termin., (001)		BO_2_	BO_2_	BO_2_	BO_2_	BO_2_	BO_2_	BO_2_	BO_2_
Layer	Ion	B3PW	B3PW	B3PW	B3PW	B3PW	B3LYP	B3LYP	B3LYP
1	B	−3.08	−1.71	−2.81	−2.25	−1.79	−1.30	−2.37	−1.38
	O	−0.35	−0.10	+0.31	−0.13	−1.70	−2.31	−1.99	−2.10
2	A	+2.51	+2.75	+5.32	+3.55	+1.94	+4.23	+4.36	+2.81
	O	+0.38	+1.05	+1.28	+0.57	+0.85	+1.25	+1.04	+0.91
3	B	-	-	-	-	−0.03	−0.05	−0.47	−0.04
	O	-	-	-	-	0.00	−0.09	−0.28	−0.05

**Table 6:** B3LYP or B3PW computed as well as experimentally detected surface rumpling *s* and respective atomic displacements Δ*d*_12_ and Δ*d*_23_ (% of *a*_0_) for the BO_2_- and AO-terminated (001) surfaces of eight ABO_3_ perovskites.

Material	Method	AO-Terminated (001) Surface			BO_2_-Terminated (001) Surface		
		*s*	Δ*d*_12_	Δ*d*_23_	*s*	Δ*d*_12_	Δ*d*_23_
BTO	B3PW [1]	1.37	−3.74	1.74	2.73	−5.59	2.51
	LDA [134]		−2.8	1.1		−3.1	0.9
CTO	B3PW [3]	7.89	−9.43	1.12	1.61	−4.46	2.75
	GGA [135]	0.37	−0.44	0.22	0.13	−0.41	0.33
PTO	B3PW [1]	3.51	6.89	3.07	3.12	−8.13	5.32
	LDA [134]		−4.2	2.6		−4.4	3.1
STO	B3PW [2]	5.66	−6.58	1.75	2.12	−5.79	3.55
	LDA [134]		−3.4	1.2		−3.5	1.6
	LEED [136]	4.1 ± 2	−5 ± 1	2 ± 1	2.1 ± 2	1 ± 1	−1 ± 1
	RHEED [137]	4.1	2.6	1.3	2.6	1.8	1.3
BZO	B3PW [82]	3.07	−4.77	0.48	0.09	−3.73	1.97
CZO	B3LYP [120]	9.22	−11.12	3.71	1.01	−5.53	4.28
PZO	B3LYP [94]	3.32	−6.26	1.04	0.38	−6.73	4.83
SZO	B3LYP [94]	6.77	−8.49	2.39	−0.72	−4.19	2.85
	LDA [138]	7.9	−9.1	3.2	−0.7	−6.1	4.2
	GGA [138]	7.8	−9.3	3.3	0.3	−7.4	4.9

**Table 7:** B3LYP or B3PW computed BO_2_- or AO-terminated (001) surface energies (in eV per surface cell) for BTO, CTO, PTO, STO, BZO, CZO, PZO, SZO perovskites.

ABO_3_ Perovskite	ABO_3_ Perovskite (001) Surface	
Termination, Functional	BO_2_-Terminated	AO-Terminated
BTO [1], B3PW	1.07	1.19
CTO [3], B3PW	1.13	0.94
PTO [1], B3PW	0.74	0.83
STO [2], B3PW	1.23	1.15
BZO [82], B3PW	1.31	1.30
CZO [120], B3LYP	1.33	0.87
PZO [94], B3LYP	0.93	1.00
SZO [94], B3LYP	1.24	1.13

**Table 8:** B3PW, B3LYP, PWGGA or HF computed Γ-Γ band gaps for eight ABO_3_ perovskite bulk as well as their BO_2_- and AO-terminated (001) surfaces (in eV).

Perovskite, Method	Γ-Γ Band Gap, Bulk	BO_2_-Termin., (001)	AO-Termin., (001)
BTO, B3PW	3.55 [114]	2.96	3.49
CTO, B3PW	4.18 [75]	3.30	3.87
CTO, B3LYP	4.20 [99]	3.33	3.88
CTO, PWGGA	2.34 [75]	2.06	2.19
CTO, HF	12.63 [75]	11.86	12.53
PTO, B3PW	4.32 [114]	3.18	3.58
STO, B3PW	3.96 [114]	3.95	3.72
BZO, B3PW	4.93 [75]	4.48	4.82
BZO, B3LYP	4.79 [107]	4.37	4.71
BZO, PWGGA	3.24 [75]	2.76	3.08
BZO, HF	12.96 [75]	12.62	12.84
CZO, B3LYP	5.40 [120]	5.22	5.00
PZO, B3LYP	5.63 [94]	4.60	3.86
SZO, B3PW	5.30 [75]	4.98	5.01
SZO, B3LYP	5.31 [94]	4.91	5.04
SZO, PWGGA	3.53 [75]	3.17	3.20
SZO, HF	13.54 [75]	13.19	13.25

**Table 9:** B3LYP or B3PW computed B-O bond populations for eight ABO_3_ perovskites bulk and also for their BO_2_-terminated (001) surfaces (in *e*).

Perovskite	Functional	B-O Chemical Bond Populations	
		ABO_3_, Bulk	Surface, (001)
BTO	B3PW	0.098	0.126
CTO	B3PW	0.084	0.114
PTO	B3PW	0.098	0.114
STO	B3PW	0.088	0.118
BZO	B3PW	0.108	0.132
CZO	B3LYP	0.086	0.102
PZO	B3LYP	0.106	0.116
SZO	B3LYP	0.092	0.114

**Table 10:** B3PW or B3LYP computed upper-layer atom shifts for 8 ABO_3_ perovskite BO-, A- and O-terminated (011) surfaces.

Term. (011)	Atom	CTO	STO	PTO	BTO	CZO	SZO	PZO	BZO
Method		B3PW	B3PW	B3PW	B3PW	B3LYP	B3LYP	B3LYP	B3PW
BO	B	−7.14	−7.69	−8.13	−7.86	−6.06	−6.16	−6.87	−6.61
	O	+4.67	+3.59	+3.30	+2.61	+4.96	+4.36	+4.27	+3.35
A	A	−16.05	−12.81	−11.94	−8.67	−18.67	−15.73	−15.17	−11.81
O	O	−6.10	−6.61	−7.37	−5.40	−5.97	−6.56	−6.61	−7.32

**Table 11:** B3PW or B3LYP computed second-layer atom shifts for 8 ABO_3_ perovskite BO-, A- and O-terminated (011) surfaces.

Term. (011)	Atom	CTO	STO	PTO	BTO	CZO	SZO	PZO	BZO
Method		B3PW	B3PW	B3PW	B3PW	B3LYP	B3LYP	B3LYP	B3PW
BO	O	−0.44	−0.51	−0.41	−1.02	−0.38	−0.38	−0.24	−0.29
A	O	+1.35	+1.02	−0.61	+0.80	+1.25	+1.24	−0.57	+0.66
O	B	−0.26	−1.02	+0.20	−0.15	+0.75	+1.45	+0.73	+0.12
	A	−2.10	−1.18	+0.18	+1.54	+1.78	−1.43	+0.73	+0.21
	O	+3.43	+1.79	+0.51	+1.95	+0.67	+4.29	+4.29	−0.78

**Table 12:** B3PW or B3LYP computed (011) and (001) surface energies for CTO, STO, PTO, BTO, CZO, SZO, PZO and BZO perovskites (in eV per surface cell).

Term.	*E*_surf_	CTO	STO	PTO	BTO	CZO	SZO	PZO	BZO
Method		B3PW	B3PW	B3PW	B3PW	B3LYP	B3LYP	B3LYP	B3PW
BO	(011)	3.13	3.06	1.36	2.04	3.46	3.61	1.89	3.09
A	(011)	1.91	2.66	2.03	3.24	1.49	2.21	1.74	2.90
O	(011)	1.86	2.04	1.72	1.72	2.08	2.23	1.85	2.32
BO_2_	(001)	1.13	1.23	0.74	1.07	1.33	1.24	0.93	1.31
AO	(001)	0.94	1.15	0.83	1.19	0.87	1.13	1.00	1.30

**Table 13:** B3LYP or B3PW computed 8 ABO_3_ perovskite bulk, BO_2_-terminated (001) surface and BO-terminated (011) surface B-O chemical bond populations in the plane (B(I)-O(I)) as well as in the direction perpendicular to the (011) surface (B(I)-O(II)).

Chemical bond	CZO	CTO	STO	PTO	BTO	SZO	PZO	BZO
Method	B3LYP	B3PW	B3PW	B3PW	B3PW	B3LYP	B3LYP	B3PW
Bulk (B-O)	0.086	0.084	0.088	0.098	0.098	0.092	0.106	0.108
(B-O), (001)	0.102	0.114	0.118	0.114	0.126	0.114	0.116	0.132
B(I)-O(I), (011)	0.138	0.128	0.130	0.132	0.130	0.142	0.148	0.152
B(I)-O(II), (011)	0.240	0.186	0.188	0.196	0.198	0.246	0.252	0.252

**Table 14:** B3LYP computed eight ABO_3_ perovskite B-terminated (111) surface three upper-layer atom relaxation (% of *a*_0_).

Layer	Ion	BTO	CTO	PTO	STO	BZO	CZO	PZO	SZO
1	B	−11.19	−6.23	−7.57	−3.58	−8.03	−11.07	−9.24	−5.72
2	A	−6.22	−14.02	−10.09	−11.24	−9.73	−11.31	+5.92	−11.92
	O	+2.74	+1.30	−0.13	+1.53	+0.78	+0.14	+2.61	+0.79
3	B	−0.25	−0.26	+0.53	+0.26	−0.02	−0.96	−2.73	+1.53

**Table 15:** B3LYP computed eight ABO_3_ perovskite AO_3_-terminated (111) surface three upper-layer atom relaxation (% of *a*_0_).

Layer	Ion	BTO	CTO	PTO	STO	BZO	CZO	PZO	SZO
1	A	−1.24	−0.52	+1.01	+1.33	+1.70	−3.61	−0.05	−0.74
	O	−3.98	−0.81	−2.52	−0.03	−0.57	−0.07	−1.26	−0.52
2	B	+2.49	+2.13	+0.02	+1.81	+0.21	+1.20	+1.18	+0.74
3	A	+1.49	+2.60	+1.26	−0.03	+0.71	−0.02	−0.02	−0.02
	O	−0.25	−0.07	+1.26	−0.26	−0.01	−0.07	−0.02	−0.18

**Table 16:** B3LYP computed surface energies for B as well as AO_3_-terminated ABO_3_ perovskite (111) surfaces (in eV per surface cell).

Termination	BTO	CTO	PTO	STO	BZO	CZO	PZO	SZO
B-terminated	7.28	4.18	6.14	4.99	7.94	8.19	6.93	7.98
AO_3_-terminated	8.40	5.86	8.11	6.30	9.33	9.62	8.21	9.45

## Data Availability

Data are contained within the article.
